# Observations on the Diseases Incident to Pregnancy and Childbirth

**Published:** 1840

**Authors:** 


### Observations on the Diseases incident to Pregnancy and Childbed. By *Fleetwood Churchill*, M.D. Dublin, 1840.

If Dr. Churchill's former work elicited the general approbation of the profession, we feel convinced, that this present continuation (not sequel we are glad to find,) will do so in a yet greater degree. It seems to us, that he has followed out his original design, of combining a condensed practical guide for the young practitioner, together with the means of pursuing the subject in all its details, for those who may wish to do so, in a much more perfect and successful manner, than he did in his former volume. The book, without pretending to contain novel views, or bold and original speculations, but rather a resumé and estimate of all that is known upon the various subjects upon which it treats, yet, is one that will be perused with great interest as well as advantage. We know of no more valuable a present to the student, nay to the practitioner himself, than the results of the careful study of medical literature, by a judicious and *practical* physician. Opinions and systems, submitted to the test of his critical powers, and accumulated experience, come before us with an authority and importance their original authors could never have invested them with, inspiring the timid practitioner with courage to continue in a course, on which, unaided, he might have felt himself unwarranted in venturing, and proving to the rash that grave errors have been committed even under the sanction of a great man's name.

To the unthinking the task of compiling such a work as the present may seem easy enough with a little industry. It is not so. Certainly, *book-makers'* compilations are as easy as abundant, (one's very stomach heaves at the idea of another being added to the huge unreadable mass), but how undiscriminating the selections, how artificial the arrangement, how ineffective the conclusions, how destitute of authority, how useless the production, and oh! how *fade* the perusal of such things. Place the materials in the experienced physician's hands, and how different the result. His opinions upon what others have advised, are nearly as interesting and as important as are the propositions he has himself to advance, and there is a freshness, a vigour, an importance, and an authority about his production, which practical knowledge can alone confer.

With pleasure then, do we proceed to present the reader with a detailed account of this valuable, learned, yet practical work.

### PART I.—DISEASES INCIDENT TO PREGNANCY.

### I. ON THE LOCAL AND CONSTITUTIONAL CONSEQUENCES OF PREGNANCY.

"Pregnancy, then, may be strictly considered as a physiological state, but as one bordering so closely upon the pathological, that it is sometimes difficult to point out the boundary between them; and not unfrequently this boundary is palpably transgressed in several organs or in their functions. In the present chapter, the changes which are induced by gestation, considered as an 'altered,' but not 'morbid' process, will be enumerated, in order that we may more distinctly appreciate the diseased actions which occasionally require our interference." 4.

The author first slightly describes the various *anatomical changes* the uterine system undergoes: thus, the loosening and enlarging the *texture* of the uterus —the great increase of vessels capable of carrying red blood to it—the altered state of the *nerves* going to it, which are not distended, but actually hypertrophied by addition to their substance,*—so too are the *lymphatics* proportionally enlarged. The organ goes on progressively encreasing, distending the abdomen and displacing the bowels, until, according to the calculations of Levret, its capacity is increased 5.19, and its solid substance in the ratio of 12 to 1, compared with the virgin state. The ovary and fallopian tube are considerably more vascular than usual.

* For an account of the recent discovery by Dr. Lee, of a large supply of nervous matter for the gravid uterus, see Medical Gazette, Vol. 26, p. 41.

That *new actions*, and new or *enlarged sympathies* should result from this altered anatomical structure, would be naturally expected; and thus, the general state of the body may become materially changed, especially by the induction of a state of *plethora*, as shewn by the usually buffy state of the blood, and increased quickness of pulse. Again, many *individual organs* suffer: some *mechanically*, and, thus, according to the degree of the enlargement of the uterus, and its position in the cavity of the pelvis or abdomen, we may have constipation, retention or incontinence of urine, severe abdominal or lumbar pain, dyspnœa, jaundice, indigestion, varicose veins, œdema, a painfully stretched or pendulous state of the skin of the belly, &c.; others from *sympathetic irritation*, as the morning sickness, diarrhœa, the production of the milky appearance in the urine (which may sometimes much assist us in our diagnosis of pregnancy), by the secretion of kiesteine by the kidneys. That the general *nervous system* should be in a very excitable state can cause no surprise, and the evil influence of depressing mental emotions and presentiments, and of disagreeable external impressions, is well known.

### II. The General Management of Pregnant Females.

In endeavouring to relieve the various evils attendant on the state of pregnancy, we must not be too meddling but content ourselves with applying a palliative treatment. Thus, although in some cases *bloodletting* will be very proper, yet, to employ this means indiscriminately, would indeed be most hurtful. In the same manner, aperients, emetics, opiates, though not to be used without good cause, may each occasionally prove of great utility, and will, indeed, together with due attention to pure air, appropriate diet, and an easy dress, enable us to combat successfully with all ordinary cases of sympathetic irritation. Even the local effects resulting from the pressure of the gravid uterus, may also be much alleviated by the use of aperients and lavements, the periodical evacuation of the bladder, and the maintenance of the recumbent or other appropriate posture.

### III. Diseases of the Genital Organs in Pregnant Women.

1. *Œdema of the Labia.*—This chiefly appears towards the latter months of pregnancy. It is usually produced by pressure preventing the return of the blood by the veins; and, according to Davis, is especially likely to occur where the pelvis is sufficiently capacious to permit the enlarged uterus to sink into it. In other cases, it may form part of a general tendency to œdema; and, indeed, in most cases the lower extremities are also thus affected. It is usually unattended by inflammation, but at other times it has coexisted with erysipelas. It may reach a large size, and must be distinguished from the sanguineous effusion occurring during labour. A mild purge and the horizontal posture are sufficient to dissipate most cases, and "much comfort is derived from bathing the parts twice a-day with tepid milk and water, and afterwards dusting them with some absorbent powder." Should the distention be great, the parts should be punctured.

2. *Pruritus of the Vulva.*—Dr. Churchill, referring to his former volume for fuller information, quotes a case from Dewees of severe pruritus, arising from aphthæ, which was completely relieved by the application of a strong solution of borax. He also recommends the use of solutions of acetate of lead or nitrate of silver, while some cases may require active antiphlogistic treatment. Dr. Dewees' case reminds us of one, which occurred to us some time since, in the person of a lady far advanced in pregnancy, who suffered the most dreadful torments from pruritus. With a delicacy we at once deplored and respected, she resisted an inspection until our stock of remedies, and her own powers of endurance were alike exhausted, when, upon examination, we found several large patches of slightly raised ulcers of a pale appearance, upon the inner surfaces of the labia. We drew a stick of nitrate of silver across each. The patient slept soundly, the first time for many a night, and scarcely ever suffered again during the rest of her pregnancy.

3. *Vaginal Leucorrhœa.*—Few pregnant women are entirely without this. It would seem to be excited by the pressure of the uterus creating irritation, and impeding the return of the blood, with which these parts are now so abundantly supplied. It is sometimes, though very rarely, accompanied by febrile action. When it is excessive it causes much debility and dorsal pain. Its removal is by no means easy, and, perhaps, of questionable propriety, seeing that it may possibly act as an useful derivative. Great cleanliness, mild astringent washes, and when the habit is feeble tonics, constitute the treatment.

4. *Mentruation during Pregnancy.*—The author refers to a large host of authorities upon this interesting subject, and afterwards thus expresses himself.

"However strange it may appear, the cases on record are too numerous, and too well authenticated, to leave us in doubt that a discharge resembling the catamenia, in colour, quality, and periodicity, does not unfrequently occur during gestation. . . . . . I have myself seen three or four cases of this deviation. In one it continued up to the 8th month inclusive; in the others it was arrested between the 4th and 6th month; but in all it was well marked, returning regularly, and varying but little in quantity and quality from the ordinary discharge. Still more remarkable and rare are those cases where the catamenia appear for the *first time* during pregnancy, or *only during* gestation." 33, 36.

The discharge is usually paler than natural, and the quantity less, but it is never coagulable. He considers its *source* to be the vaginal mucous membrane, aided perhaps by that of the cervix.

5. *Discharge of Watery Fluid from the Vagina.*—This is quite distinct from leucorrhœa, being a colourless bland fluid, discharged in quantities varying from a few ounces to some pints daily, by which, however, the size of the abdomen is not lessened. Great weakness and lumbar pain accompany it. Dr. Churchill is in doubt whether the vaginal mucous membrane, or the chorion be its source. Dr. Davis calls it a dropsy of the chorion. Every accoucheur must be familiar with examples, in which there has been dribbling of considerable quantities of fluid from the vagina, for days, or even weeks, prior to labor, and yet at that time, the membranes are found apparently perfect. Dr. Davis considers the affection a dangerous one, but our author does not take so serious a view of it. It is not under our control.

6. *Dropsy of the Amnion.*—Dr. C. justly observes that as this results from an excessive action of the secretory vessels of the amnion, so, properly speaking, it should be considered a disease of the ovum, and not of the uterus. It is sometimes connected with a diseased state of the placenta. Its chief symptom and inconvenience arises from the excessive distention of the abdomen. The child is usually feeble or diseased at birth, or it dies before. Labor may be temporarily delayed by it, and flooding is more likely to follow. All we can do is to attend to the well-ordering of the general system.

"Should the distention be enormous, and the distress be very great, we shall be justified in having recourse to the induction of premature labor, especially because in these cases the child is generally lost when left to nature. Whilst this operation is in our power, it appears to me quite unjustifiable to have recourse to abdominal paracentesis, as recommended by some authors (Scarpa, Desmarais, Davis.)" 51.

7. *Rheumatism and Spasm of the Uterus.*—Although rheumatism of the uterus is recognised as a well-established disease by several German and French writers, yet we agree with the opinion formerly expressed in this journal,* that, although the possibility of such an affection occurring in a muscular tissue like the uterus may be admitted, yet, that the cases hitherto published are by no means conclusive. So, also, Dr. Churchill, by placing this disease under the same head with spasm of the uterus, would seem to doubt its identity with rheumatism, as no one can assert more than a very partial similarity between the two diseases. Indeed, whether we consider the symptoms he enumerates, or the means of cure he recommends, (consisting of occasional depletion, sudorifics, and opiates) we see rather the signs of spasmodic affections, accompanied sometimes with a little fever, than of rheumatism. The simultaneous existence in some cases of pains of a rheumatic character in other parts of the body, is almost the only fact in favour of the existence of the latter.

8. *Hysteritis.*—This affection is usually limited to the portion of the uterus where the placenta is attached; the cervix, however, often eventually participates in the diseased action, while the rectum and bladder become sympathetically excited. There is often much constitutional disturbance. If the disease be extensive the child will probably perish in utero, or be prematurely expelled. Dr. Murphy† states, that rupture of the uterus may often be traced to hysteritis, occurring during gestation. Leeches in sufficient number, and the affecting the mouth slightly with calomel and opium, are the usual means of cure.

---

* Med. Chir. Rev. No. 62, p. 563, and No. 63, p. 238.
† Dublin Journal, Vol. 7.

### IV. DISORDERS FROM SYMPATHETIC IRRITATION.

### (*a. The Chylopoietic Viscera.*)

1. *Tooth-Ache.*—When this is a neuralgia, produced by the irritation of the uterus, creosote, local stimuli, or counter-irritation, may be employed. Gardien gives stramonium, and Blundell the volatile tinct. of valerian and carbon. of iron. When the gum is inflamed, leeching, scarifying and purgatives: it may be even necessary to bleed. When depending upon catarrh, sialagogues, as pyrethrum, tobacco, or a stimulating gargle, are useful. When caries is present, and the usual means unsuccessful in removing the pain, Dr. C. recommends extraction, providing no disposition to abortion exists.

2. *Salivation or Ptyalism.*—In the salivation of pregnancy, the gums are not spongy or ulcerated, nor is there any fætor present. When slight it is of little consequence, but when in excess, and sometimes many pints are spit daily, the stomach becomes very much debilitated. The patient however does not suffer to the extent that might be supposed, and many authors prohibit all attempts to check the discharge. Indeed, this is no easy matter to accomplish, but, when the patient's digestive powers become enfeebled, it should be attempted by counter-irritation behind the ears, aperients, and mild astringent gargles.

3. *Fastidious Taste and Capricious Appetite.*—From the time of Mrs. Pickles' celebrated *longings* for the scarce pine-apples, and for her neighbour's porcelain pôt-de-chambre, husbands and physicians have been often tormented in endeavouring to appease these cravings. Sometimes we suspect, as in the above-named lady's case, they result from capricious or obstinate humour, but, in the great majority of cases, they arise from a morbid state of system, whether this be dependent upon mere deranged sympathy between the stomach and the uterus, or whether it be dependent upon actual disorder of the digestive organs themselves. "But a more remarkable peculiarity, and one less explicable, is the depravation of appetite we sometimes meet with, when the patient utterly repudiates articles of diet of which she was previously fond, or acquires tastes repugnant to her previous habits, or even to common sense." Dr. Churchill, though utterly repudiating the idea of "mother's marks" affecting the fœtus, yet believes that this disordered state of the digestive and nervous system of the mother may prevent the due nourishment of her offspring. True kindness consists in resisting, not in gratifying these desires, when extravagant or depraved. The diet and state of the digestive organs should be carefully attended to. In some cases depletion, baths, antispasmodics, anodynes, &c., according to circumstances, have been found useful; and these fancies usually disappear about the fourth month.

4. *Nausea and Vomiting.*—Vomiting usually occurs in the morning, and ceasing to be troublesome about the second or third month, seldom comes under the physician's notice, yet, in other cases, it may come on during various periods of the day, and is sometimes so violent as to cause abortion. Again, when it constantly occurs after meals, the patient may sink, worn out by constant irritation, and deprivation of nutrition. The milder cases result from a mere sympathy with the gravid uterus, but the more obstinate depend sometimes upon a state of plethora, or in other cases, as many facts tend to prove, upon a morbid state of the womb itself. Carus assigns a congested state of the portal system as another cause. The stomach, in many cases, may become secondarily inflamed. In the *treatment* of these cases, we should interfere very little in moderate examples, which, indeed, by the removal of indigestible articles of food, may act beneficially, while mere *nausea* will often be effectually relieved by a gentle emetic. Most authors are agreed, that the cases dependent on plethora, are best relieved by small occasional bleedings (which according to Dr. Campbell are most useful at the menstrual periods), or, where this is not admissible, by the application of leeches to the epigastrium. Aperients, counter-irritants, effervescing draughts, the local or general use of opium, have each at times been found useful: the same may be said of Prussic acid, inf. calumb., mint tea, iced water, and where there is acidity, charcoal, alkalis, or the mineral acids. The *diet* is a most important point, and it should be of the lightest possible description, and given only in the smallest quantities. Dr. Ashwell has found very small quantities of milk, when combined with a little lime or soda water, or a few grains of calcined magnesia, remain on the stomach better than any thing else. If the affection resists all these means, and the patient's life is eventually likely to be placed in danger, the induction of premature labour is to be recommended.

5. *Heartburn or Cardialgia, Pyrosis.*—Although usually arising from sympathy with the uterus, these may be caused or aggravated by various descriptions of food, and they may also arise from mental emotions, and a deranged state of the bowels. The appetite is usually destroyed, but otherwise, except in very severe cases, there is not often much general suffering. The *treatment* consists in attention to the *diet* and state of the digestive organs, and in the administration of various alkalis; where these do not suffice, antispasmodics, opium, the abstraction of a little blood, and mild bitters, may each be required under varying circumstances.

6. " *Cramps of the Stomach and Duodenum.*—Under this title Dr. Burns has described an affection not very uncommon with pregnant females. It consists of a cramp-like pain in the region of the stomach and duodenum, occasioning considerable suffering, and even sometimes causing abortion. It is probably dependent upon the state of the bowels, or may be caused by errors in diet, or mental emotion. In some few cases it would appear to be connected with the passage of a biliary calculus, and may give rise to jaundice. Occasionally, however, it is a less simple affection, being complicated with congestion of the head, threatening convulsions, and accompanied with tenderness of some portion of the spine." 108.

In *treating* these cases, after having relieved the pain by a full dose of laudanum, we must regulate the bowels by means of aperients. In the intervals, tonics and counter-irritation at the epigastrium. In severe attacks, accompanied by congestion of the head, depletion, with counter-irritation at the epigastrium, may prevent an attack of convulsions.

7. *Hæmatemesis.*—This occurs sometimes, though very rarely, in the early months of pregnancy, but is attended with little danger. Attention should be chiefly directed to the state of plethora which usually produces it.

8. *Constipation.*—This may depend upon both the pressure exercised by the uterus, and the altered state of the vitality of the bowels. Great accumulation sometimes occurs without much uneasiness, and may be overlooked, owing to the passage of a small quantity of liquid stool daily; but generally, both the local and general irritation are considerable, and abortion has even arisen from the tenesmus and repeated ineffectual attempts to empty the bowel. Every practitioner is aware of the difficulties and dangers that may arise from these accumulations during and subsequent to parturition. In *treating* the complaint we must bear in mind, that the bowels are naturally somewhat confined during gestation, and, therefore, we must not be too active in the use of purgatives. Very mild aperients, assisted by warm water enemata, answer best; yet, in obstinate cases, the bowels must be cleared out by more active purgatives, or even relieved, in old cases, by mechanical means.

9. *Diarrhœa.*—This, though sometimes independent of, at others precedes or co-exists with constipation, and would seem to be occasionally vicarious of the menses. Generally a slight affection, in other cases, it is of so aggravated a character as to endanger abortion or the life of the mother. It is not always judicious to stop it, but it may be kept under by alkalis and mild astringents, or by small doses of hydrarg. c. cretâ and pulv. ipecac. co. (an admirable combination for all analagous affections of the alimentary mucous membrane) and the opiated starch glyster. Frequent, irritating, yet small stools are best relieved by a dose of castor oil, containing 30 drops of laudanum. Burns recommends small doses of ipecac. and rhubarb. Some cases may require bleeding, leeching and blistering, while, in all, the diet and dress should be particularly attended to.

10. *Jaundice.*—This usually comes on towards the termination of gestation, owing generally to the pressure of the uterus upon the gall-ducts, and arising, in other cases, from sympathy with the gravid uterus, or from disease of the liver or gall-bladder. In some of these latter cases, great constitutional irritation is present, and may be followed by abortion, or even the death of the woman. The state of pregnancy obliges us to modify the means we might otherwise employ, but, yet, moderate depletion in the plethoric, the cautious use of the blue pill and anodynes, accompanied by occasional purgation, must be had resort to.

"Some females acquire a dark, almost yellow color of the skin during pregnancy, which must be carefully distinguished from the disease in question, as it is of no consequence, requiring no treatment, and disappearing after delivery."

(*b. Circulating System.*)

11. *Palpitation of the Heart.*—Most females suffer more or less from this during pregnancy, but especially those of a nervous temperament. The respiratory and nervous systems become affected, giving rise to many morbid sensations, and it is often connected with hysteria. In its *treatment* we must pay much attention to posture, bleed if plethora exist, and give antispasmodics, opiates, or tonics, to the feeble and delicate. The regulation of the diet and the state of the bowels is of great importance.

12. *Syncope.*—This is not invariably confined to the period of quickening. In delicate persons it sometimes comes on periodically. It is said to be inducible by the opposite states of plethora and anæmia. Burns has met with fatal cases, in which the heart has been found to be diseased. Frequent syncope is probably injurious to the child, and is sometimes followed by abortion. Its occurrence during labor, is always an unpleasant, and sometimes an alarming occurrence, but it does not seem to retard its progress.

(*c. Respiratory System.*)

13. *Dyspnœa* may arise from various causes; hysterical women are very liable to it in the early months, from sympathy with the gravid uterus, the attack in them being short, sudden, and without fever. In the middle months it seems to be connected with a congested state of the lungs, producing sometimes pneumonia or pulmonary apoplexy, and, of course, accompanied by great constitutional disturbance. In the latter months, it is produced by the pressure of the enlarged uterus, occurring especially in first pregnancies. The treatment required is much the same as if pregnancy were not present.

14. *Cough.*—This is often very obstinate and irritating, destroying rest, and producing head-ache, spasm, and sometimes abortion. In the early months, it is of a spasmodic nature, and unattended with expectoration. In the latter months, it may arise from the congested state of the lungs, produced by the pressure of the uterus upon the aorta, and against the diaphragm, or from a degree of bronchitis when it is attended with febrile action. In some circumstances, bleeding, counter-irritation, and aperients are required; in others, antispasmodics, anodynes, mild expectorants, and diaphoretics.

15. *Hæmoptysis.*—In the few cases where this is found to occur, it generally comes on during the early months, probably owing to the recent suppression of the menses. Capuron observed that most of those subject to it had malformed chests. Treatment not peculiar.

(*d. Nervous System and Senses.*)

16. *Sleeplessness.*—This most distressing affection is by no means uncommon in the latter months, affecting especially, as do all the inconveniencies of pregnancy, the nervous and hysterical. Convulsions occasionally result. Some women fortunately can compensate for their loss of rest by a little sleep in the day, and the restorative effect of a little so taken is sometimes remarkable; distressing and fatiguing dreams often render her sleep of little benefit to the woman. We must endeavour to calm the nervous irritation which usually exists, by the use of mild laxatives and anodynes, by the use of the pediluvium at bed-time, and even, if required, by the detraction of a little blood. Attention to diet, free exercise and fresh air are important, while, to the very weak, tonics may be cautiously given.

17. *Hypochondriasis or Despondency.*—The brain becomes so peculiarly affected, by sympathizing with the pregnant state, that, we find a woman sometimes afflicted with a settled despondency, without obvious cause or anticipated danger. It is nowise surprising that the victim of seduction should be remarkably liable to this dejection. It usually diminishes as gestation advances, but, at other times, may proceed to actual insanity. The progress of labor may be materially influenced, as may the subsequent "getting up," by this unfortunate state of mind. There is often much headache, and many symptoms of derangement of the digestive organs.

*Treatment.*—In the slighter cases, time, the arguments of friends, and the due regulation of the bowels will gradually remove the affection, but in the graver cases the state of the brain must be attended to, and, in the words of Burns, "if the despondency be preceded by excitement, marked by heat of skin and frequency of pulse, or by congestion at the base of the brain, marked by slow pulse, and feebleness or languor, venesection will be proper, and in determining this, no attention is to be paid to the paleness of the visage." The author thus concludes the subject.

"As to the *moral* treatment, I have always found that a fair and honest statement concerning the suffering and danger in prospect, has far more effect than an attempt to make light of the case. By admitting her expectations of considerable suffering to be true, we are more likely to gain credit with her than when we insist upon the risk being very slight." 155.

18. *Head-ache* usually of a nervous character during the early months, during the latter depends on plethora, and may, if not relieved, lead to convulsion. The character of the pain and accompanying symptoms, vary according to the different sources of the head-ache, and so must the treatment. The digestive organs are often much deranged.

19. *Convulsions.*—To prevent repetition, the author treats in this place both of the convulsions which precede and succeed labour. He thinks, that much of the contradictory opinion existing among medical men, arises from the want of a due classification of the various cases: he follows that of Dewees, and therefore treats successively, of hysterical, epileptic, and apoplectic convulsions.

(*a.*) *Hysteric Convulsion.*—This occurs only during gestation, and usually comes on in the early months. Dewees considers the contraction of the muscles of the back into an arched form to be pathognomic of it. It is not usually a very alarming occurrence, though occasionally premature labor has come on during a paroxysm. There is not the same degree of convulsive movement, the complete insensibility, the frothing of the mouth, or stertor, seen in epilepsy; while, after the fit is over, the patient returns to her natural state. Generally, antispasmodics and diffusible stimuli are required, rather than means of a lowering character, while, a refreshing sleep may often be obtained after the paroxysm by a dose of opium.

*(b.) Epileptic Convulsion.*—The sanguine temperament, and especially in first labors, is most liable to this, the most frequent form. In 38,306 labors, epilepsy occurred 79 times, or once in about 485. It would seem to be produced, in some cases, by the brain sympathising with various sources of irritation; and in others, by the accumulation of blood produced in that organ during labour. The symptoms are those of ordinary epilepsy. The paroxysms are sometimes very frequent, and as many as eighteen in the twenty-four hours have been known to occur. Instead of imperfect recovery from the attack, a state of complete coma may sometimes result, or the patient may even have become maniacal.

When these convulsions occur *during pregnancy*, they generally come on during the latter months, and it is seldom that the patient goes the full time. The child is still-born, often putrid, while the mother usually does well. Convulsion hardly ever occurs in cases of malpresentation.

*During labor* the paroxysm comes on with the commencement of a pain, but not with every pain, and all the symptoms are more intense than during gestation. If not terminated by art, the labor usually runs its natural course, and is by no means necessarily fatal to the infant, although it is in great danger. These cases are frequently followed by abdominal inflammation after delivery.

When they occur *after delivery*, they usually come on in from two to four hours, and may succeed to labors perfectly natural. The loss of blood, by flooding, &c. at the time of delivery, does not prevent the fits, but adds to the debility.

*Prognosis.*—"I should say that the cases where the convulsions occur during labor, and continue afterwards, are the least manageable; next to these, the attacks during labor only; then, those after delivery; and, lastly, the most favorable are those which occur during gestation." Out of 152 recorded cases, 42 ($\frac{1}{4}$) mothers were lost. After recovery, the patient may enjoy her usual state of health, and is not very liable to a recurrence of the disease in subsequent labours.

*Treatment.*—The first indication is free bleeding, and thus even from 40 to 70 ounces have been taken. It may require to be repeated in more moderate quantities, or leeches and cupping substituted. A strong purge will evacuate the bowels, and may accelerate the uterine contractions. The head should be shaved, and cold applied to it, while, after a time, active counter-irritation must be resorted to. The propriety of giving *opium* has been much canvassed, and Dr. Churchill thinks it may be administered advantageously after due depletion. Calomel has been found useful, when given to affect the gums, and Dr. Collins speaks highly of the utility of nauseating doses of tartar emetic.* The propriety of interfering with the

---

* After bleeding and purging, Dr. Collins gives a table-spoonful of the following mixture every half-hour:—℞. Aquæ pulegii ℥viii. ant. tart. gr. viii. tr. opii gt. xxx. syr. ʒii. M. In some cases, four grains only of tartar emetic were put in the mixture, whilst, in others, the quantity of laudanum was encreased.

progress of labor is a most important question. Dr. Churchill entirely disapproves of the idea of producing forcible dilatation, and is opposed also to the operation of turning; but he considers we certainly should apply the forceps, when the head has descended low enough, or the perforator, when this is not the case, providing the safety of the mother requires peremptorily that delivery should be completed.

(c.) *Apoplectic Convulsion.*—This seldom, if ever, occurs, except towards or after the termination of labor, being caused by the stress upon the cerebral vessels during the labor-pains. It is usually preceded by headache, flushing, &c. The convulsive movements are usually very brief, after which the patient falls into a comatose state, accompanied by stertorous breathing. There is no partial return of intelligence, and rarely more than one paroxysm. The pulse is slow and full, while the pupils are insensible to light. "In almost all cases, the condition of the patient remains unaltered until death; but there are a few cases, answering, I presume, to the congestive apoplexy of Abercrombie and Lallemand, where our timely aid is successful, and the patient recovers sense and motion; and, if proper care be taken, is speedily well." Upon examination after death, blood is usually found poured out into the ventricles, at the base, or into the substance of the brain. The most active depletion should be immediately instituted; and, in apoplexy resulting from congestion, relief may soon follow, but the other cases are nearly hopeless.

20. *Nervous Affections of the Ears and Eyes.*—Delusions referable to these organs of sense are frequent, depending, when they are not purely nervous, upon a state of congestion of the brain, or of the organs themselves. In some cases the sense becomes lost altogether. When the affection is nervous, counter-irritation, tonics and antispasmodics, with due regulation of the bowels, are required; but where evidence of congestion is present, bleeding, general or local, must be resorted to.

*(e.) The Breasts.*

21. "*Mastodynia.*—In ordinary cases, about the second month, the patient's attention is directed to the breasts, in consequence of a sensation of pricking, tingling, or shooting pain in them, accompanied with increase in size, and a degree of soreness of the nipples. If the breast be grasped, it will be found to have lost its peculiar softness, and to have acquired a firm glandular consistence: the gland encreases as pregnancy advances, until it seems to constitute the entire substance of the breast, the fatty tissue having nearly or altogether disappeared. This disappearance of the softer tissue is often very remarkable. Imbert speaks of a patient of his, whose breasts, large before conception, always decreased during pregnancy in consequence of it." 193.

Usually, little inconvenience attends these changes, but, in other cases, severe suffering may result from various causes, as the firmness of the fibrous capsule resisting the encrease of the gland, congestion, or inflammation, neuralgia, &c. Fomentations, poultices, or anodyne frictions, may be applied. Narcotics may be required. Where inflammatory action is present local or general depletion is useful, and nauseating doses of tartar emetic particularly.

V. Disorders arising from Mechanical Pressure or Distention.

1. *Herniæ* may be protruded between the fibres of the distended recti, or by reason of a partial separation at the linea alba; or, again, through the enlarged natural openings of the abdomen. Strangulation is likely to occur when the hernia is old, and has contracted adhesions; and, therefore, in all cases of inordinate constipation or vomiting during pregnancy, we should always carefully examine the abdomen. The treatment is obvious.

2. *Hæmorrhoids.*—These occur less frequently in first than in subsequent pregnancies, and, in many women who have borne many children, delivery does not rid them of the evil:—the distended veins never recovering their proper size. In some, they only come on after delivery, owing to the pressure exerted during labor. Mild aperients, tepid or anodyne enemata, leeching, with a bland diet, usually give considerable relief. Dr. Ashwell recommends the use of a tepid lotion (dec. papav. ℔.j. liq. pl. ac. ℥j.) to allay the irritation after a confined motion, as also the injection of a few ounces of warm olive oil, twice daily. He says, much relief is derived from pressing upon each individual pile, until its cavity becomes emptied of blood. After the irritation is subdued, various astringents may be used.

3. *Spasm of the Ureters.*—Burns attributes to this cause the occurrence of severe pain in the region of the ureters and kidney, accompanied by distressing stranguary. Purgatives, enemata, opiates, and counter-irritation, form the means of cure.

4. *Incontinence of Urine.*—This distressing affection frequently torments pregnant women in different degrees, and produces sometimes great local and constitutional irritation. It arises, in the early months, from a morbid irritability of the neck of the bladder, or from a temporary paralysis of this same part, produced by the pressure of the uterus upon it: in the latter months, the uterus exerts this same pressure upon the fundus, producing a degree of paralysis also, which is sometimes not relieved until long after delivery. In the *treatment* of the former class of cases, depletion may be sometimes required, while in the others, fomentations, accompanied by narcotics and demulcent drinks, will suffice. When produced mechanically, little is to be done; cold sponging the back sometimes encreases the retentive powers of the bladder. The patient must also anticipate the involuntary by a voluntary discharge of her urine; always, likewise, paying great attention to the bowels.

5. *Dysuria, Retention of Urine.*—Denman says retention of urine occurs only in natural labor. If neglected, it will produce retroversion of the womb, while the most serious injury may befal the bladder, if permitted to remain distended during labor. According to circumstances, depletion, anodyne fomentations, &c. may be required; and, where the affection has resulted from diminished sensibility and over-distention of the bladder, efforts should be made at intervals, and cold applications applied to the vulva. When the retention is complete the catheter must be used.

One word, *en passant*, as to *passing the catheter* in the female. Simple as this operation usually is, difficulties sometimes occur during and after labor, when the parts are distorted from their natural situations, or concealed by tumefaction. First, then, the accoucheur will find a middle-sized gum elastic male catheter, (better still if it were constructed of the flattened form of female catheters,) used without the stilette, a far more manageable instrument than the silver catheter in common use: from its flexibility, it will follow the tortuosity of the canal, and may be passed, even when the head has descended pretty low, without risk of rupture, while, owing to its length, it can be much more easily made to communicate with a basin held at the edge of the bed. Again, after labor, it is a far better plan not to pass the finger from before backwards, to feel for the orifice of the urethra; but, placing the forefinger at once in the vagina, and feeling the round cord at its upper part, denoting the urethra, the catheter may be usually slid along this finger with the greatest ease into the orifice.

6. *Cramps and Irregular Pains.*—These often cause great suffering, and are more frequent about the fourth or fifth months, and at the end of gestation, owing to the then greater pressure upon the nerves, which, together with the distention of the muscular and ligamentous structures of the abdomen, is the chief cause of their occurrence. When situated in the lower extremities, they often destroy the security of the woman's footing. As they depend on mechanical causes, little is to be done. We must pay great attention to position, and the state of the digestive organs. Some few cases require the loss of blood, while anodynes and counter-irritants may also be tried—especially the spt. terebinth. A belladonna or opium plaister is also sometimes useful.

7. *Varicose Veins.*—These seldom occur during a first pregnancy; sometimes, though rarely, the veins of the labia, vagina, and even of the os uteri, are affected, as well as those of the lower extremities. Although usually subsiding after delivery, the varicose state may continue sometimes in women who have borne many children for years, while others are only first affected with varicose veins after delivery. Recumbent position, bandages, and the prevention of loaded bowels are all the means of palliation we possess.

8. *Œdema and Anasarca.*—Affecting usually only the ankles, sometimes the hips and vulva are involved, or even a general state of anasarca may exist. Arising, usually, from mechanical causes, at other times it would seem to depend upon a plethoric or atonic state of the system. It usually disappears immediately after labor, but, when it is complicated with erysipelas, it becomes a much more serious affair. Rest and the recumbent posture are usually sufficient, in the slighter cases; and, where great distention is present, Gardien recommends punctures to be made. Erysipelas, or any inflammatory affection, must be treated on general principles.

9. *Ascites and Hydrothorax.*—These cases, except when they result from organic disease, are almost always examples of acute inflammatory dropsy, and occur usually towards the end of gestation. In some cases the children have been born affected with ascites, in others healthy. The diagnosis of ascites and pregnancy combined must be carefully made, to distinguish it from the former alone, and the prognosis should be very guarded. As a general rule, in extreme cases, Dr. Churchill prefers the induction of premature labor to the performance of paracentesis.

### Part II.—DISEASES INCIDENT TO CHILDBED.

### Chap. I.—On the Management of the Convalescence of Puerperal Women.

To save repetition, we shall embody under this one head our analysis of the author's three chapters, on the ordinary convalescence after parturition —the management of puerperal females—and, the variations from ordinary convalescence.

#### *(a.) The State of the Nervous System. The Nervous Shock.*

"The sudden alteration of the eye, the diminished or increased sensibility of the brain, the disturbance of the respiratory and circulating system, the altered secretions, the great exhaustion, &c. all are evidence of a shock to the nervous system, the effects of which are thus extensively felt. After easy labors it is not very remarkable, and the patient soon recovers from it; but it is too manifest to be questioned after those of a more serious character. It has been usual to attribute the exhaustion of the patient to the fatigue resulting from muscular effort; but when the whole of the immediate consequences of labor are considered, and especially when extreme cases are examined, I think there is proof of much more than muscular exhaustion." 241.

The effects of this shock, when it is moderate, soon subside, and, in proportion to its slightness, the patient becomes quickly restored to a state of comfort. To ensure this, quietude of mind and body, as far as possible, must be maintained, so that sleep, or at all events that passive tranquillity of the system, which is its best substitute, may, if possible, be procured.

But the shock may be *severe.* In such cases:—

"The patient complains of great exhaustion; the senses are either unnaturally dull, or morbidly acute; the breathing is hurried or panting, and the accordance between the respiration and circulation is broken off. The aspect of the patient is that of a person in a state of collapse. The countenance is expressive of suffering, anxiety, and oppression. The pulse may be either very slow and laboured, or unusually rapid, very small, and fluttering. There are many cases, however, where the shock, though far from being so severe as in the case I have supposed, is quite sufficiently so to excite the fears of the medical attendant. Reaction is long before it occurs; or it may take place imperfectly or excessively, and the patient remain for some time in a very weak condition." 259.

In such cases, the patient may recover gradually, or death may occur in a few hours, leaving no traces of disease discoverable after death. The best remedy in these cases is *opium*, giving the patient a chance of salutary sleep, or at all events tranquillizing the agitated state of the nervous and circulatory systems. The very careful use of moderate stimuli may be allowed:—ammonia or musk, combined with opium, being the best, although even wine or brandy may be required. Suckling must be deferred, or, if necessary, entirely abandoned.

(*b.*) *State of the Circulation and Respiration. The Pulse.*

" I have carefully investigated the state of the pulse in a number of cases; and in the majority I have found the following alternations take place. During the second stage of labor, the pulse always encreases in frequency, though the amount varies in different persons. Shortly after delivery it falls nearly, but not quite, in proportion to its former frequency ; *i. e.* it becomes nearly as much below the ordinary standard, as it was above it, previously. After the lapse of a few hours, a reaction takes place, the amount of which is nearly, but not quite in proportion to the original increase and subsequent collapse. Again, after 12 or 14 hours it subsides, to be again encreased on the secretion of milk; after which, if the patient go on well, it gradually returns to its ordinary standard. To illustrate my meaning, let us suppose that during the second stage the pulse mounts to 120 ; then, during the collapse, it will fall perhaps to 60 ; and, on reaction taking place, will rise to 100 or 110. I do not intend to give this illustration as the accurate standard of these changes, but merely as illustrative of the alternations I have generally observed; nor do I say that they occur in every case, but only that I have noticed them in a very large majority." 242.

There is no better index of impending mischief than an abnormal state of the pulse, and it should therefore be narrowly watched. The author agrees with Dr. Clarke, that no woman can be considered safe whose pulse continues more than 100. Besides the varieties in the pulse, in severe nervous shock, already alluded to, the author has remarked another peculiarity, in cases of *flooding*, viz. its *quickness.*

" In almost all the cases of flooding after labor, when I have had an opportunity of examining the pulse up to the time of the occurrence, I have found it remain quick, and perhaps full, instead of sinking after delivery. This has been so marked in several cases, that I now never leave a patient so long as this peculiarity remains ; and in more than one instance I believe the patient has owed her safety to this precaution. Three cases occurred within a very short time of each other, in which I noted this undue quickness of the pulse without any other untoward symptom; at that time there was no excessive discharge, and the uterus was well contracted. In all these, alarming hæmorrhage occurred within an hour, and was with difficulty arrested.

I have also remarked an undue frequency of pulse when the *after-pains* are extremely violent; and as the uterus is in such cases rather tender on pressure, it requires care to distinguish between this state and the commencement of puerperal fever." 261.

It may also become accelerated by lactation, the retention of coagula, diarrhœa, or gastric disturbance.

*(c.) State of the Uterus, Vagina, &c.*

The uterus, contracting immediately after labor, in some hours after enlarges again, and then again gradually diminishes in size, until the 8th or 9th day, when it sinks into the pelvis. His description of the *interior of the uterus* is very good.

" The condition of the cavity of the uterus is of great interest. When examined a day or two after delivery, the lining membrane appears loose and corrugated, somewhat softened, and covered more or less by patches of the the decidua. The part to which the placenta was attached, is raised above the level of surrounding parts; its surface is unequal, resembling in this respect a granulating ulcer; its size is wonderfully reduced. The whole internal surface is of a dark ash colour, while the discharge upon it may be greenish or brownish, giving the appearance of a morbid condition of the parts—indeed I have known it pronounced gangrene. The structure of the uterus, if cut into, is found to be less dense than natural, and the fibres more distinct. The sinuses are still very evident, and at the placental situation they are filled with clots of blood. The os and cervix are covered with ecchymoses, as though they had been severely bruised; and sometimes small lacerations may be observed in the edge. The orifice remains open for some days, but gradually closes." 244.

The *vagina* is reduced to its natural dimensions in an incredibly short period of time. Some hours after labor the vulva should be washed with a little tepid milk and water, containing a little spirits, and this should be repeated daily, in order to maintain the parts cleanly, and to aid in restoring them to their natural state. The integuments of the *abdomen* remain flaccid and pendulous for a long time, if the patient have not been properly bandaged. The author insists upon the importance of the *bandage*, both in effecting the above object, and in securing and maintaining good uterine contraction. We believe few cases of flooding ever occur where this is attended to, from the moment of delivery. But, it must not be left to the nurse, who is usually too stupid a personage to apply it either in the right manner, or the right place. Where a proper stay is not at hand, the best substitute is formed by passing a broad firm towel, quite low down, and bringing it tightly over the hips, upon a pad formed of one or two napkins rolled up, previously placed just over the uterus, and which prevents the bandage rising up, by filling up the hollow between the ossa ilii. The author also deems the *horizontal posture* very important, as suited to the relaxed state of parts after delivery, and as even sometimes aiding the replacement of old displacements. We agree with him here too, but, we think it quite unnecessary, in ordinary cases, to keep the woman *in bed*, after the second or third day, when the little feverish action, generally accompanying lactation at first, has passed away. Both her strength and comfort, and probability of sleeping well at night, are encreased by letting her be outside the bed, or on a sofa, during the day.

(*d*). *After-pains.*—Although the presence of coagula generally exasperate these, yet may they occur just as severely when there are none. Within certain limits they are undoubtedly salutary. Sometimes they are of an agonizing severity, and may be accompanied by a quick pulse and tender abdomen, which may throw some doubt upon their nature; but, the tenderness is greatest during the existence of the pains and subsides with them. A full dose of laudanum (not less than 40 drops) should be given, and soon repeated, while fomentations are applied to the belly. We have also found severe after-pains yield best to this treatment, generally giving 60 drops for a dose, and following it up, in a few hours, with an active purge; ordering, as long as the pain lasts, flannel bags filled with hot salt, to be continually applied—a description of fomentation we find much more easily applied, and much less liable to be neglected, than any other we have tried. In a great number of patients, who had suffered most severely before from after-pains, we have succeeded in procuring often nearly an immunity from them, by the use of the draught copied below,* for the first 24 hours after labor, beginning soon after the child is born, and following its employment by a purge.

*(e.) The Lochia.*—Sometimes this ceases naturally in a few days, especially in women who have borne still-born or putrid infants; but generally, in our climate, it may continue about a month, more or less, according to the constitution of the individual. Nurses and patients attach a vast, and unnecessary importance to the various variations in this discharge. Sometimes it is too profuse in quantity, or too prolonged in duration, causing great debility. If the patient get about too soon, the red colour, or even secondary hæmorrhage may come on. The fætor, though sometimes of an insupportable character, is no indication of disease of the uterus.

*(f.) The Secretions and Excretions.*—During labor the secretion of the *skin* is much encreased, and even after delivery continues more free than usual, giving it a soft greasy feeling. Thus, although we should not expose our patient to a draught of cold air, yet, on the other hand, she must not be oppressed by too many bed-clothes. Notwithstanding the perspiration, the secretion of the *kidney* is usually encreased after delivery also. The patient should endeavour to pass water six or eight hours after delivery. "Owing to the distensible state of the abdominal parietes, the patient will often wait much longer if not reminded; and the consequences may be very troublesome, if not serious. The bladder may become paralysed, or inflammation may spread from it to the peritoneum." A cloth wrung out in warm water and applied to the vulva, will often relieve a difficulty of passing water.

The state of the *bowels* varies, but, generally speaking, a more or less confined state requires the use of aperients† in twelve or fourteen hours.

*(g.) State of the Breasts.*—Varieties in the period of secretion, and, indeed, a total absence of it, are not usually attended with any ill consequence. We cannot subscribe to all the directions in the following extract:—

"When the breasts begin to enlarge, and to be painful, warm fomentations, frictions with warm oil, or a slightly stimulating liniment, may be employed. A dose of purgative medicine should be given. It is better to put the child to the breast, even if it should not be the intention of the mother to suckle her infant, (?) as it will afford relief, and by not suffering the child to do more, we ensure the ultimate subsidence of the secretion, which is always in proportion to the demand upon it, and if this be very slight, it will soon cease altogether." 257.

*Inflammation of the Breast.*—This is especially frequent after first labors,

---

* ℞. Liq. ammon ac. ℥ss. Liq. ant. tart. ♏x. Tr. hyoscy. ♏x.—xx. Syrup. aur. ʒj. Aquæ m. v. ℥j. M. ft. ht. cap. j. 4tis horis.—*Rev.*

† We have found an aperient mixture, recommended by Dr. Conquest, peculiarly useful after delivery. ℞. Pulv. jalap. ʒss. mag. sulph. ℥ss. tr. zingib. ʒss. aq. m. p. ℥iii. M. ℥j. vel. ℥jss. pro dos.—(*Rev.*)

and if not soon relieved matter forms. A slight affection, when merely the skin and cellular membrane beneath it are affected, but causing intense suffering when the gland itself and the fascia become affected. In the latter case, in a person of bad constitution, or when it has been badly treated, sinuous burrowing abscesses are formed, draining the patient's strength, and sometimes placing her life in danger. In *treating* such cases, after depleting generally or locally, purgatives, cold lotions, fomentations, or poultices must be made use of. Tartar emetic is especially useful. When matter has formed we must make an incision, as soon as fluctuation is felt, and not wait for the pointing of the abscess.

*Sore Nipple* is more frequently found in the first labor ; the suffering from this cause is sometimes dreadful. To *prevent* this disorder the author recommends there should be applied to the nipples, during the last months of pregnancy, spirit and water night and morning; we have found a solution of alum particularly useful. For the *treatment*, he recommends astringent lotions or ointments, especially the nitrate of silver; and, if the irritation be not overcome, the shield must be had recourse to. Every accoucheur knows the troublesome nature of these cases, and the occasional difficulty in curing them. We would recommend that women, not accustomed to suckle, should not apply the child to the breast *too frequently ;* their doing so being often the cause of the affection appearing. They believe that the child's crying is always a sign that it wants the breast, and thus sometimes give it to it twenty times a day, besides letting the little creature drag at them all night. Suckling, under such circumstances, is often a martyrdom, productive of broken rest and disordered health ; we have long been in the habit of pressing upon mothers the propriety and facility of establishing, from the first, a habit with the child of expecting the breast only at certain hours—say six or eight times in the twenty-four hours. By this means, the mother's nipples will be often preserved unbroken, and her health unchanged, while the child will be saved from those disastrous effects upon the digestive organs, which a constantly replete state of the stomach would even yet more frequently produce, were not the milk generally rejected by vomiting soon after suckling.

(*h.*) *Diet.*—Dr. Churchill recommends continuing the slops until the third day, and allows a little meat only on the seventh or eighth day. This is rather too rigorous. When the woman is going on well, and the slight feverish reaction usually consequent on the establishment of lactation has passed, we may let her gradually assume her usual diet, limiting only quantity. Indeed, it is better to mention some simple article of animal food as soon as possible, or the woman, when left to her own invention, and often stimulated by a good appetite, will be very apt to indulge, *sub rosa*, in stewed eels, or some such " nourishing " mess.

2. *Sanguineous Tumor of the Labia.*—This rare affection, coming on during labor sometimes extends even into the pelvis or to the perineum, and at others contains six or seven pounds of effused blood, the exact source of which is by no means very clear. The tumor may rupture, and thus give relief spontaneously ; but this is followed in some cases by fainting or death.

When the blood has had time to coagulate in the swelling, we should make an incision into the tumor (applying a compress if any fresh bleeding occur,) and remove the effused blood.

3. *Inflammation of the Vagina.*—This may occur after tedious or preternatural labor. From this cause sometimes portions of the mucous membrane slough away, and cicatrices are formed most inconveniently for future sexual connexion and parturition. In treating these cases we must employ the usual antiphlogistic means, especially tartar emetic. During the healing of the sores contractions should be prevented, by the daily maintaining in the vagina, for a short period, gradually encreased bougies, of which a wax or tallow candle form the best.

4. *Puerperal Fever.*—We shall not pursue this subject at great length; not that it is not of paramount importance, or that the author has not produced a most learned and excellent account of it, but because he has brought forward no new facts upon the subject, and that it has been very fully entered into in a recent number of this Journal.*

Dr. Churchill thinks five varieties are distinguishable, for, although these may be often blended more or less together, yet in the various epidemics, the prominent local affection has generally been sufficiently distinct. The varieties are:—

(*a.*) *Puerperal Peritonitis.*—This, which has characterised many epidemics, would seem to attack the peritoneum of the uterus primarily, and then spreading to the other portions of the same membrane, often involves the uterine appendages. Severe after-pains with fever, intestinal irritation, and hysteritis have their several diagnostic marks which distinguish them from this disease.

(*b.*) *Inflammation of the Uterine Appendages.*—This seldom exists alone, being usually accompanied with peritonitis—the distress, however, being more referable to the iliac regions. Exhaustion is also more likely to ensue rapidly upon the inflammatory symptoms. Bleeding is obliged to be employed much more carefully than in the former variety, and leeches must often be substituted for it.

(*c.*) *Puerperal Hysteritis.*—Inflammatory action, affecting the substance of the uterus, is by no means rare. Tonnellé found it in 188 cases out of 222—Dugès, in three out of four—and Lee, in ten out of forty-five cases. *Softening* is a frequent consequence of it, and the place of attachment of the placenta is an usual situation of it. Here, too, exhaustion occurs sooner than in puerperal peritonitis. The mild cases usually recover by moderate depletion, calomel and opium, and counter-irritation; the severe cases defy our resources.

(*d.*) *Uterine Phlebitis.*—Tonnellé found pus in the veins in 93 out of 134

* Med.-Chir. Rev. No. 60, p. 496.

cases, and Lee marks of phlebitis in 24 out of 45 cases. The latter believes, besides the usual causes of puerperal fever in general, that this form may arise from mechanical injury inflicted on the uterus, either during labor, or by the extraction of the placenta. The pain is generally more limited and local than in peritonitis, but the diagnosis is often obscure in the early stages ; while in the latter, the nature of the disease is elucidated by the production of various diseased actions, accompanied usually by purulent depositions in remote organs, as the brain, lungs, heart, joints, &c. Severe and epidemic cases defy our resources, and, in all, it is necessary to have a speedy recourse to stimuli.

(e.) *Inflammation of Uterine Lymphatics.*—This too has been observed by several French writers, and by Dr. Lee.* The local symptoms are very severe, and the constitutional ones identical with those of uterine phlebitis.

5. *Rupture of the Uterus and Vagina.*—This is a most elaborate chapter. The accident has occurred 65 times in about 42,768 patients, that is, once in 657. It rarely happens in a first pregnancy. The uterus may be ruptured during labor in consequence of a diseased state of its tissue having existed during gestation,† or on account of the narrowed state of the upper outlet of the pelvis, or again, according to some, from the oblique position of the uterus, directing the child's head against the side of the cervix. Sometimes one of the component structures of the uterus, as the peritoneal, or muscular, has been torn without other portions suffering, but the accident is just as dangerous. In several cases the os uteri has been completely torn off during labor,‡ and, indeed, whenever it occurs from mechanical causes, the vagina is usually involved. The circumstances which should excite our fears, are the occurrence of hysteritis, imperfectly cured, during gestation, and the coincidence of very violent labor-pains with a narrow pelvis. There is always more or less blood effused, which may lead to peritonitis. Rupture of the uterus may also occur *during gestation* from hysteritis, accidents, or without obvious cause ; and, again, it may occur, independently of pregnancy, at an *advanced period of life*, arising from a process of absorption or thinning of the uterine parietes, produced by distention from the uterine mucus or other fluids being debarred their natural exit, owing to the thickened state of the os uteri, reducing or obliterating the passage. The principal *symptoms* are—the occurrence of a most dreadful cramp-like pain—the sense of something giving way, accompanied sometimes by a noise audible to the patient—the suspension of the labor-pains—hemorrhage and rapid collapse —the presentation no longer to be felt from the vagina, providing the rupture be complete.

In *treating* these cases the first question is as to delivery, and, wherever the os uteri has not been in an undilated state, all experience has been in favour of this being performed instantly ; for, in nearly all cases of recovery, delivery has been accomplished. The forceps, or perforator, according to circumstances, must be used, and, when the child has escaped into the abdomen,

* Medico Chirurg. Trans. vol. 15, p. 64. † Murphy, Dublin Journ. vol. 7.
‡ Medico Chir. Trans. vol. 11, and Dublin Journal, vol. 16, p. 54 and 154.

we must follow it through the rent into that cavity, or, if this cannot be done, the Cæsarian section may be had recourse to, which also affords the only means of treating rupture of the uterus occurring during gestation.

6. *Vesico-vaginal Fistula.*—These accidents, so deplorable in their effects upon the woman's comfort, are not rare. Some one of the following circumstances may give rise to them: wounds and injuries of the vagina by instruments, pessaries, &c. or from the pressure of the child's head—too prolonged retention of urine—laceration of the bladder, &c. The following rule is a good one.

"In all cases, a careful examination should be made, by passing the catheter into the bladder, and a finger into the vagina; then placing the points of both in apposition, the whole posterior surface of the bladder should be passed over, and carefully examined.* At some one point the finger and catheter will come in contact: the catheter may then be passed into the vagina, and the extent of the damage ascertained." 384.

We will not follow the author in his long and interesting detail of the various means which have been tried for the *cure* of these dreadful cases. Indeed, upon this point we are almost in despair, having witnessed how little success attended the persevering efforts of the late lamented Mr. Earle, who brought to the task all that a sympathising humanity, unwearied patience, great mechanical skill, and a profound acquaintance with the powers and resources of nature could furnish. However, much may be done for *palliation* of this disgusting and distressing infirmity, and we will extract the author's description of some of the means.

"Dr. Gooch, in 1814, suggested to Mr. Barnes the employment of an India-rubber bottle, of sufficient size to fill the vagina, and having upon one side of it a small piece of sponge, to be applied to the fistulous opening. Mr. Barnes used this with great benefit to his patient."—*Med. Chir. Trans.* Vol. 6, p. 586.

"Dr. Evory Kennedy has succeeded in taking casts (with wax) of the vagina with the fistula, in several cases; and from them he made moulds, and had caoutchouc bottles cast in the moulds. These were large enough to fill the vagina, and to close the outer opening, so as entirely to prevent the escape of urine.

I have attained the same object by means of a piece of sponge covered with thin bladder. It should be large enough to fill the vagina and of a suitable shape. A narrow neck, of the dimensions of the vaginal orifice, is to be formed, by wrapping it with twine, which is to be covered with lint. The whole has much the shape of an egg-cup. It should be dipped in oil previous to being used, and then it can be easily introduced, and the stalk filling up the external orifice, no urine can escape. It can be removed and replaced by the patient herself." 400.

7. *Laceration of the Perineum.*—This is most likely to happen in first

---

* "This is the more necessary, inasmuch as a temporary incontinence of urine is not uncommon after delivery. It generally also comes on soon after labor, so that at first either may be mistaken for the other. A vesico-vaginal examination will always enable us to distinguish them. This incontinence, which arises from a species of paralysis of the bladder, is best treated by the frequent evacuation of the urine—rest—and, when the lochia have ceased, by cold bathing."

labors. Its *extent* is very various; thus, the rent may extend only to the sphincter ani, or it may involve it; in other cases the rupture may take place centrally, having the fourchette and the sphincter untouched; again, the posterior part of the sphincter may be torn open into the rectum, leaving the anterior part of the plane of the perineum untouched.

The *causes* of the accident enumerated by the author are numerous. Thus the *sacrum* may be too *perpendicular*, so that the head will be forced down upon the posterior part of the perineum; so also, if the *arch of the pubis be too acute*, preventing the head fitting into it, it will be forced with more than ordinary violence against the perineum; the *too rapid progress of the head*, either on account of the violence of the pains, or from its too small size, will throw it too directly upon the perineum, without its having received the requisite changes of position during its passage through the pelvis: *excessive breadth of perineum* may produce the accident, by letting the head rest on the centre, instead of gliding towards the anterior edge; again, *rigidity* of the perineum, an old *cicatrix, pelvic exostosis*, a *weakening* of the perineal tissue by previous disease, an occlusion of the *hymen* may each be a cause, as also may *malposition of the child's head*, by presenting a too long diameter. *Præternatural* presentations, by not adapting themselves to the configuration of the parts, are more likely to cause the rupture than head-presentations. The *awkward position* of the woman, her *starting* away, or employing too strong *bearing-down* efforts, may each contribute to the production of this disaster. Lastly, it may be torn by *instruments*, which should, as a general rule, be removed just before the head passes through the vaginal orifice. We copy the author's valuable directions for the—

"*Preventive Management.*—1. Defects in the passages, which render the mechanism of expulsion inefficient, may often be remedied by the application of the hand in such a manner as to give a direction forward to the head. 2. Direct support should be given to the perineum when distended; but this is frequently *carried to excess*, and produces the accident it is intended to prevent; it should be moderate and gentle—just so much as to support the parts and no more. I must altogether object to any attempt to retard the passage of the child as erroneous in theory and mischievous in practice. 3. When the perineum is rigid and undilatable, benefit may be derived from fomentations with hot water, the use of warm oil, lard, or pomatum. 4. Under no circumstances is it justifiable to dilate the external orifice with the hand, as formerly recommended; on the contrary, instead of drawing back the perineum, it ought to be carried forward. 5. If laceration be threatened in consequence of the persistence of the hymen, it may be incised with a blunt-pointed bistoury. 6. The patient should always cease forcing, and remain perfectly quiet during the exit of the child." 408.

As to the means to be employed for the *curative* treatment we must refer to the work itself, merely observing that slight cases require little or nothing to be done.

8. *Phlegmasia Dolens.*—Although this may occur after a first labor, it is more frequent in women who have borne several children. Delicate women are especially liable to it, and particularly if they have suffered from uterine irritation during or after labor. After taking an historical survey of the various opinions as to the *nature* of phlegmasia dolens, the author thus expresses his own.

" It is evident, that if we take pathological anatomy for our guide, we must conclude the disease to consist in inflammation of the veins of the lower extremities, in many cases propagated from the veins of the uterus; and that the interruption of the circulation through these vessels gives rise to the effusion in the cellular tissue. This view also derives some support from the phenomena which result from phlebitis in other situations. At the same time it is not impossible that some further information may be necessary, before we fully comprehend the true nature of the disease." 419.

We think the author's *prognosis* is rather too favourable.

" Though we cannot say that the disease is without danger altogether, when severe, yet the proportion of deaths is so small, that in the great majority of even severe cases, our prognosis may be favorable: still more decidedly when the attack is slight." 425.

*Treatment.*—Depletion is to be performed rather by leeches than by the lancet; while cathartics, combined with tartar-emetic, are often useful. There is much difference of opinion as to the propriety of blistering the extremity; some, as Dewees, condemning the practice, while others consider it a specific;* opiates must be employed if required, and the blandest diet insisted upon. After the acute stage has passed, gentle support, counter-irritation, and tonics are desirable.

Dr. Churchill, we think, altogether takes too light a view of the danger of this disease. He has not sufficiently alluded to those examples of it, in which typhoid symptoms rapidly replace the acute inflammatory stage, requiring a much more early and active exhibition of stimuli and tonics, than would be proper in a common simple case. These cases are especially likely to occur in the epidemic constitution of the atmosphere, which favors the occurrence of puerperal fever, to which indeed they are nearly allied.

9. *Puerperal Mania.*—Of the mania occurring *during* labor, the author thus speaks:—

" The temporary delirium, or mania, which occurs during labor, was, I believe, first recorded by my friend, Dr. Montgomery.† It appears at two particular periods of the labor—first, as the head passes through the os uteri, and again, at its exit through the os externum. It would appear to be owing to the extreme suffering at these times, acting upon an irritable and nervous temperament. It is very temporary, generally lasting but a few minutes, and then subsiding. The most curious point about it is, that the patient is generally *conscious* of her incoherence. A lady whom I attended a short time ago, and in whom this delirium occurred, assured me that she knew she was talking nonsense, but that she could not resist it." 430.

Puerperal mania, *after delivery*, is not rare. It usually comes on before the secretion of milk is established, but it may appear much later, nay, even as the result of weaning. The phenomena do not differ from those seen in ordinary insanity. There are two classes of cases, in one of which a quick pulse and fever are present, another in which they are not. In *treating*

---

* See Wyer, London Med. Phys. Journal, 134; and Edinb. Med. Surg. Journ. vol. 15, p. 156. Sankey, Ed. Journ. vol. x. p. 402.

† Dublin Journal, vol. v. p. 51.

these cases, depletion must be very cautiously employed. The most benefit seems to be derived from cleaning out the bowels by purgatives and enemata, and then administering an opiate, when the pulse does not forbid. Antispasmodics and diffusible stimuli, combined with opiates, have also proved useful. When the pulse is very quick, small doses of tartar-emetic are of great benefit. The greatest quietude and attention to diet must be attended to, and eventually tonics will be required. Waller recommends the child should cease sucking. The moral treatment requires great judgment; but, generally, more good is got by seeming to humour, than by resisting the patient.

We do not doubt that long before this, the reader has been convinced of the justice of the opinion we pronounced upon this work in commencing our analysis. Although this analysis is a very full one, it has by no means exhausted the treasures of the book, and every one, who wishes to make himself master of the subjects upon which it treats, will do well to obtain it. The arrangement is a little faulty, but that is a subject hardly worth naming in a work of this kind.

